# Resveratrol Attenuates Malathion Induced Damage in Some Reproductive Parameters by Decreasing Oxidative Stress and Lipid Peroxidation in Male Rats 

**Published:** 2019-06

**Authors:** Cyrus Jalili, Shiva Roshankhah, Mohammad Reza Salahshoor, Mohammad Mehdi Mohammadi

**Affiliations:** 1 Medical Biology Research Center, Kermanshah University of Medical Sciences, Kermanshah, Iran; 2 Department of Anatomical Sciences, Medical School, Kermanshah University of Medical Sciences, Kermanshah, Iran; 3 School of Nursing and Midwifery, Kermanshah University of Medical Sciences, Kermanshah, Iran

**Keywords:** Resveratrol, Reproductive Parameters, Malathion, Oxidative Stress, Lipid Peroxidation

## Abstract

**Objective:** Malathion is the most organophosphates which capable to produce free radicals and induce disturbance on some of male reproductive parameter. Resveratrol is an herbal polyphenol and it has been beneficial antioxidant effects during short-term administration. This study was designed to evaluate the effects of Resveratrol against damage induced by Malathion to the reproductive parameter of male rats.

**Materials and methods:** In this experimental study, 48 male rats were randomly assigned to 8 groups: normal control (saline) and Malathion control (250 mg/kg) groups; Resveratrol groups (2, 8, 20 mg/kg) and Malathion + Resveratrol (2, 8, 20 mg/kg). Treatments were administered intraperitoneally and gavage daily for 65 days. The sperm parameters, testis malondialdehyde (MDA), total antioxidant capacity (TAC), testosterone level and germinal layer height were evaluated and statistically analyzed.

**Results:** The results displayed that the values of all parameters except MDA level (which increased) reduced significantly in the Malathion control group compared to the normal control group (p < 0.001). The Resveratrol and Resveratrol + Malathion treatments at all doses increased significantly all parameters except MDA level (which decreased) compared to the Malathion control group (p < 0.001). No significant modifications were observed in all Resveratrol groups compared to the normal control group (p > 0.05).

**Conclusion:** Resveratrol attenuates toxic effect of Malathion on some of male reproductive parameters.

## Introduction

Human infertility is a highly critical process that is influenced by many factors such as parents’ age, maternal conditions, smoking, alcohol and coffee consumption, socioeconomic status, genetics, hormonal imbalance, and pesticides (1). Swan et al. showed that pesticides might elevate male infertility (2). Occupational exposure to pesticides and its detrimental effects on male infertility cause delayed pregnancy without contraceptive use, miscarriage, stillbirth, reduced birth weight, and growth disorders (3). Malathion is an unsystematic organic phosphorous compound that exists in a yellow to dark brown oil belonging to the family of organophosphates (4). This toxin is extensively used in agricultural fields and gardens to destroy pests. The reduced weight of sex organs, reduced motility, increased abnormality, and sperms deaths have been reported due to administration of organophosphates (5). Fortunato et al. showed that Malathion could induce the production of free radicals and oxidative stress and increase the activity of antioxidant enzymes (6). In normal conditions, there is an imbalance between the elimination and production of free radicals in the body of living organisms. Imbalance in these processes causes oxidative stress, which can cause serious cell damage when it is intense or prolonged (7). Antioxidant enzymes are responsible for detoxification of free radicals. Catalase and superoxide dismutase are the key enzymes of this system. Further, glutathione and thiol are the most frequent non-enzymatic intracellular antioxidant (8). Organophosphates are able to change the antioxidant system of cells, cause membrane lipid peroxidation, and induce cell membrane damage via production of free radicals (9). Increased lipid peroxidation and production of free radicals from metabolism of organophosphates have been proposed as the main mechanisms involved in the impairment of cells and body tissues (10). Organophosphates can affect the sperm chromatin structure by changing the phosphorylation of the protamines of the nucleus and exerting negative effects on sperm viability, motility, and morphology, especially in the final stages of maturation (11). Organophosphates are alkylating agents. Alkylating agents can influence spermatogenesis, alter the sperm chromatin structure by bonding to protamine and DNA, and cause sperm degeneration (12). Resveratrol is a plant-derived polyphenolic phytoalexin that is produced by stilbene synthase enzyme in response to environmental stresses (13). Resveratrol exists in at least 72 plant species and fruits, especially grape skin (50-100 µg per 1 g wet weight) (14). Resveratrol inhibits inflammation through direct inhibition of the activity of COX-1 and COX-2 (15). Resveratrol can reduce the production of E2 prostaglandin and ROS from lipopolysaccharides of activated microglial cells via suppressing the activity of NF-Kappa β and I-Kβ kinase (16). Resveratrol has inhibitory effects on free radicals, possesses antioxidant properties, and increases a number of anti-oxidative enzymes. The antioxidant ability of this polyphenol is dependent on the properties of its polyphenolic hydroxyl groups (17). Given the antioxidant effects of resveratrol, it seems that this material can protect the male reproductive parameters against Malathion -induced oxidative damage. A review of the literature shows no study has evaluated the effects of resveratrol against Malathion -induced oxidative stress in reproductive parameters of male rats. Therefore, this study was aimed to determine the effects of resveratrol against Malathion -induced oxidative stress in the reproductive parameters of male rats.

## Materials and methods

***Animals: ***This experimental study was done on 48 male Wistar rats (weighing 220-250 g) at Kermanshah University of Medical Sciences. All animals were treated in accordance with guidelines of National Institute of Health for the Care and Use of Laboratory Animals approved by Research Deputy at Kermanshah University of Medical Sciences based on WMA Declaration Ethic of Helsinki (Ethic number; IR.KUMS.REC.1397.306). The rats were maintained on a regular diet and water ad libitrum with a 12:12 h light/dark cycle at 23°C ± 2°C in animal room of medical school of Kermanshah University of Medical Sciences by considering 1-week adaptation prior to the experiment (18).

***Study groups and treatment of animals: ***The rats were randomly divided into 8 groups (n = 6), including; 1: First group, the normal control group, which received normal saline (intraperitoneally injection) equivalent to the amount of experimental groups. Second group, the control group of Malathion, in this group, the rats were given Malathion at a dose of 250 mg/kg (1/50 LD50) body weight per day (single dose) through gavage (the solvent of Malathion was normal saline). Third to fifth groups, the Resveratrol administration groups, in these group, each animal respectively received (2, 8 and 20 mg / kg) of Resveratrol intraperitoneally for 65 days at 10 am. Sixth to eighth groups, Resveratrol + Malathion administration groups, in this group, each animal received single dose (250 mg / kg) of Malathion via gavage in order to induce reproductive parameters damage, then they respectively received (2, 8 and 20 mg / kg) of Resveratrol intraperitoneally for 65 days at 10 am (13, 19).

***Animals' dissection and sampling*** ***: ***At the end of the treatment period, all rats were deeply anesthetized by intraperitoneally injection of ketamine HCl (100 mg/kg) and Xylazine (10 mg/kg). Blood was taken from the heart without cutting the chest. The samples were kept in a 37°C incubator for 20 minutes and then centrifuged at 255 g in 15 minutes. The blood serum was isolated and part of the serum was kept at -70 °C for evaluating TAC, nitrite oxide and testosterone levels. Then, the chest and abdomen of animals were then cut, respectively. The epididymis tail was isolated from the testes and placed in DMEMF12 / FBS5% culture medium. The left testis was removed from the abdominal cavity and fixed in a 10% formalin solution for histological and morphometric examinations and the right ones for the MDA level estimations, in the respect of the groups (20).

***Sperm cells collection: ***Both cauda epididymides from each animal were crushed and conserved in a warmed petri dish containing 10 ml Hank’s balanced salt solution at 37°C. The spermatozoa were allowable to disperse into the buffer. After 15×^18^).

***Progressive motility: ***In this method, four degrees of sperm motility were studied based on WHO methods, class A: progressive motility. Progressive motility of the sperm cells of each sample was examined by an optical microscope with a magnification of 40 in 10 fields of view. For this purpose, first, about 50 μl of Semen liquid culture medium was taken and placed on a slide culture that was previously cleaned and dried with alcohol. Then the slide culture was placed on it and examined under the microscope. Sperm cell counting was performed through a cell count device, and about 100 sperm cells were counted in each sample. In all experimental and control groups, the count was repeated (13). 

***Survival rate: ***In this method, eosin staining was used to identify living sperm cells from dead sperms. The basis of this staining is the absorption of stain by the membrane of dead cells and its disposal by the membrane of living cells. At the end of the given time, about 20 μl of the medium containing semen fluid was collected from each dish, then mixed with an equal volume of eosin stain solution (about 20 μl). After about 2 to 5 minutes, part of the mixture was poured onto a neobar slide culture. Then, living sperm cells lack stain and dead sperm cells become pink. The prepared slide culture was examined with magnification 40 ×. At least 100 sperm cell were calculated from each random sample from the 10 fields of imagining and the percentage of live sperm cell was documented (20). 

***Sperm cells morphology: ***The normal sperm cells morphology was assessed through examination of sperm smears from the right cauda epididymis. An aliquot of the sample was used to make the smears to appraise the malformations in the spermatozoa. Eosin/nigrosine stain was used to guesstimate the normal spermatozoa morphology. One drop of eosin stain was added to the suspension and mixed slightly. The slides were then observed underneath a light microscope at 400× magnification. A total of 400 spermatozoa were studied on respectively slide (4000 cells in each group) for irregularities of the head and tail (13). 

***Sperm calculation: ***To analyze the quantity of sperm cells, 400 μL of the sperm suspension was diluted through formaldehyde fixative (Sigma; USA). Approximately, 15 μL was removed from the diluted solution into a haemocytometer by a Pasteur pipette. The haemocytometer was located into Petri dish with dampened filter paper and allowed to stand for 10 min. The stable sperms were counted and assessed per 250 small squares of the haemocytometer using a ×40 objective. The amount of sperm per mm^3^×^2^×^20^). 

***The tissue preparing and staining for evaluation of germinal layer seminiferous tubules: ***The non-parenchymal tissues (fat, fascia and vessels) of removed left testis was dissected and preparing paraffin embedded blocks were gotten using Automatic Tissue Processor. The steps of this process was consequently included fixation with 10% formal saline (for 72 hours), washing thoroughly under running water, dehydrating by raised a doses of ethanol (50, 60, 70, 80, 90 and 100%, which included 3 min for each step and 100% ethanol step was repeated for three times), clearing by xylene (three times and 10 min in each), and embedding in soft paraffin (three times and 15 min in each). At this stage, 5- µm coronal histological thin sections were cut from paraffin-embedded blocks, undertaken by a microtome instrument (Leica RM 2125, Leica Microsystems Nussloch GmbH; Germany), and 5 sections per animal were chosen. For the unification of the section selection, the first section was the 4th and the last was the 24th (5 sections interval) and finally, the routine protocol for Hematoxylin and Eosin staining was implemented. At the end of tissue processing, the stained sections were mounted by entalan glue and assessed under microscope Olympus BX-51T-32E01 research microscope connected to a DP12 Camera with 3.34-million pixel resolution and Olysia Bio software (Olympus Optical Co. LTD, Tokyo, Japan) (18).

***Testosterone*** ***measurement: ***The collected blood was centrifuged at 23°C for 15 minutes with 5000 g to get the serum. The serum samples were then kept in deep freezer (-18^o^C). The serum testosterone level was examined through ELISA (Abcam 108666, USA) technique (13).

***Measurement of renal malondialdehyde: ***MDA levels in right testis tissues were evaluated as an index of lipid peroxidation. In this regard, homogenizing of the samples were carried out by homogenization buffer containing 1.15% KCl solution and the specimens centrifuged at 1,500 g for 10 min, respectively. Then, the homogenated subjects were added to a reaction mixture containing SDS, acetic acid (pH 3.5), thiobar-bituric acid, and distilled water. Following boiling the mixture for 1 h at 95°C and centrifuging at 3000 g for 10 min, the absorbency of the supernatant was measured by spectrophotometry at 550 nm light length (21).

***Estimation of renal total antioxidant capacity: ***To measure the TAC, an acquisition kit (Cat No: TAC-96A) ZellBioGmbH-Germany was purchased, which was the basis for the oxidation colorimetry resuscitation. The kit contains 1 reagent ready to use, buffer X 100, dye powder, reaction suspension solution, standard and a microplate of 96 wells. In this assay, the TAC was equivalent to some antioxidant in the sample that was compared with ascorbic acid as standard. The kit's sensitivity was equal to 0.1 mM and the diagnostic range was mM 2-125 / 0, and final absorbance was read at 490 nm and unit conversion was performed (21).

***Statistical analysis: ***After extracting the information, Kolmogorov–Smirnov test was first conducted to confirm the data compliance of the normal distribution. The data were analyzed by SPSS software for windows (version 20) using one-way ANOVA postulation followed by Tukey’s post hoc test, and P < 0.05 was considered significant. The variables were represented as mean ± standard error of mean.

## Results

***Progressive sperm motility and sperm cell viability: ***Malathion caused a significant reduction in the sperm cell viability and progressive motility compared to the normal control group (p < 0.001). No significant variations were detected in Resveratrol groups comparison with normal control group (p > 0.05). Also, sperm cell viability and progressive motility in all treated Resveratrol and Malathion + Resveratrol groups increased significantly compared to the Malathion control group (p < 0.001) (Table 1).

**Figure 1:**
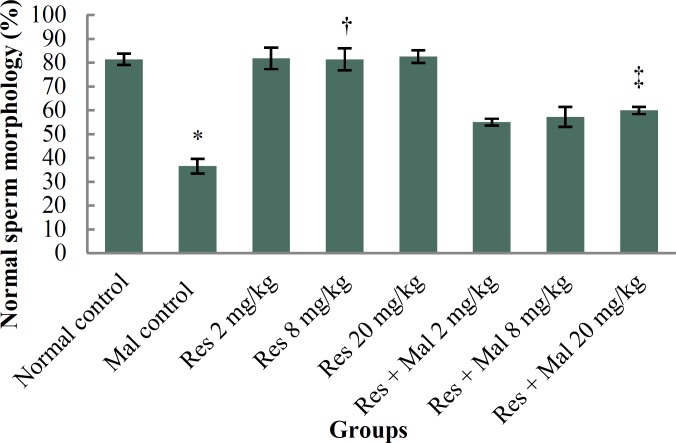
Comparison of normal sperm cell morphology in treatment groups.

***Sperm cells count and normal morphology: ***The sperm cell count and morphological normality reduced significantly in the Malathion control group equated to normal control group (p < 0.001). No significant deviations were experiential in the Resveratrol groups in contrast with the normal control group (p > 0.05). However, the sperm cell count and normal morphology were enhanced significantly in all treated Resveratrol and Malathion + Resveratrol groups compared with the Malathion control group (p < 0.001) (Figure 1 and Table 1).

**Table1:** Effect of Malathion, Resveratrol and Resveratrol + Malathion on sperm parameters in male rats (n = 6 for each group).

	**Mean of sperm count (10** ^6^ **)**	**Sperm progressive motility (%)**	**Sperm viability (%)**
Normal control	85.37 ± 1.06	19.6±1.32	75.53±1.16
Mal control	31.16 ± 4.05*	1.87±1.40*	40.83±3.05*
Res 2 mg/kg	85.75 ± 2.43†	21.12±1.21†	76.62±2.09†
Res 8 mg/kg	86.12 ± 5.07†	20.87±1.74†	76.55±5.04†
Res 20 mg/kg	85.25 ± 4.07†	20.50±0.67†	75.05±1.07†
Res + Mal 2mg/kg	49.50 ± 2.50¶	7.12±1.33¶	54.35±5.08¶
Res + Mal 8 mg/kg	51.36 ± 3.17¶	8.87±1.51¶	56.37±2.09¶
Res + Mal 20mg/kg	55.25 ± 4.23¶	8.75±1.10¶	57.21±3.51¶

Data are presented as mean ± SEM.

* p < 0.001 compared to the normal control group.

† p < 0.001 compared to Malathion control group.

¶ p < 0.001 compared to the Malathion control group. Rse: Resveratrol; Mal: Malathion

*Significant increase compared to the normal control group (p < 0.001). †Significant increase compared to the Malathion control group (p < 0.001). ‡Significant increase compared to the Malathion control group (p < 0.001). Rse, Resveratrol; Mal, Malathion.

**Figure 2:**
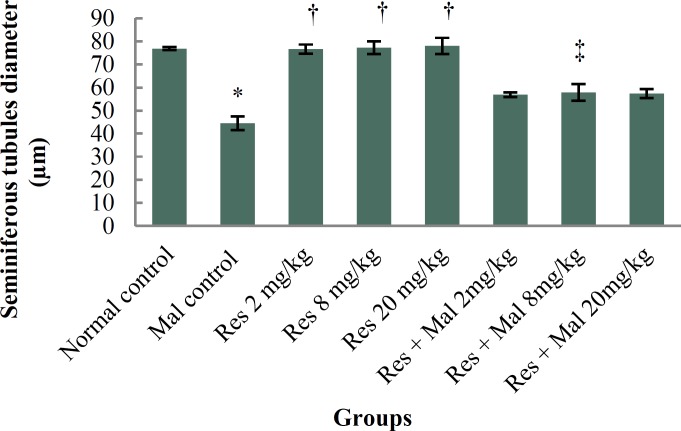
Comparison of germinal layer seminiferous tubule height in treatment groups.

**Figure 3:**
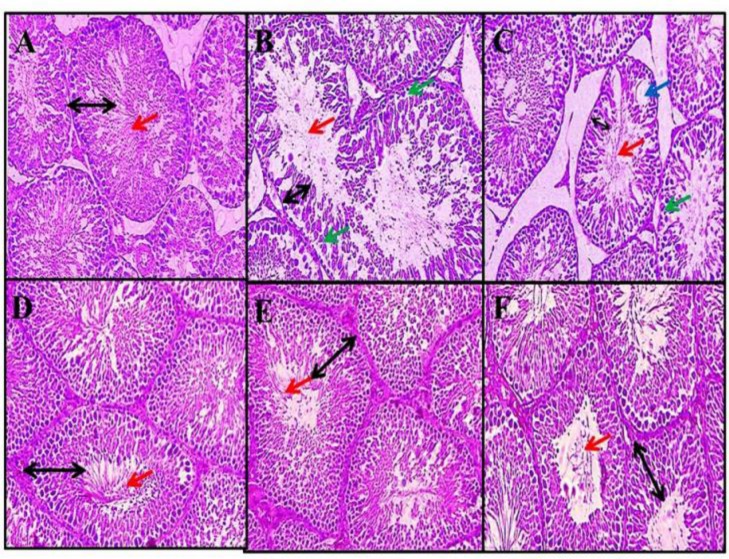
Effect of Malathion Resveratrol and Resveratrol + Malathion on seminiferous tubules (magnification ×400).

***Germinal layer of seminiferous tubules height: ***Malathion caused a significant reduction in the germinal layer of seminiferous tubules height in comparison with the normal control group (p < 0.001). No significant alterations were witnessed in comparison with normal control group (p > 0.05). Germinal layer of seminiferous tubule height in entirely treated Resveratrol and Malathion + Resveratrol groups improved significantly compared to the Malathion control group (p < 0.001) (Figure 2 and Figure 3).

*Significant decrease compared to the normal control group (p < 0.001). †Significant increase compared to the Malathion control group (p < 0.001). ‡Significant increase in compared to the Malathion control group (p < 0.001). Rse, Resveratrol; Mal, Malathion.

**Figure 4:**
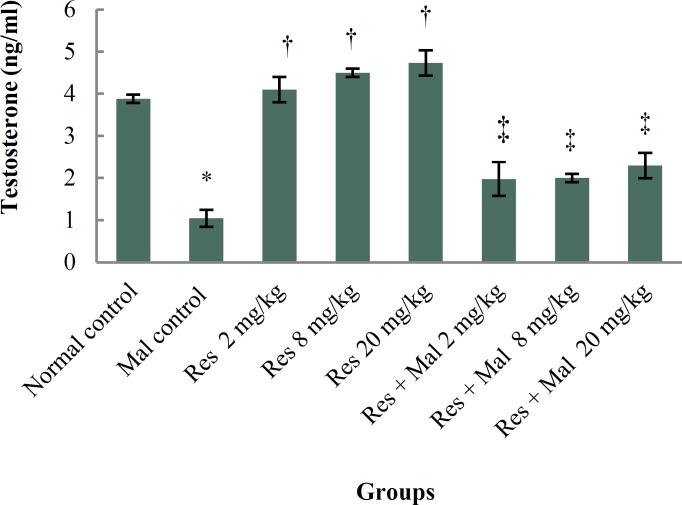
Comparison of testosterone hormone level in treatment groups.

***Testosterone*** ***: ***Malathion caused a significant decrease in the testosterone hormone level compared to the normal control group (p < 0.001). No significant alterations were detected in Resveratrol groups comparison with normal control group (p > 0.05). Furthermore, testosterone hormone level in all treated Resveratrol and Malathion + Resveratrol groups improved significantly compared to the Malathion control group (p < 0.001) (Figure 4).

**Figure 5:**
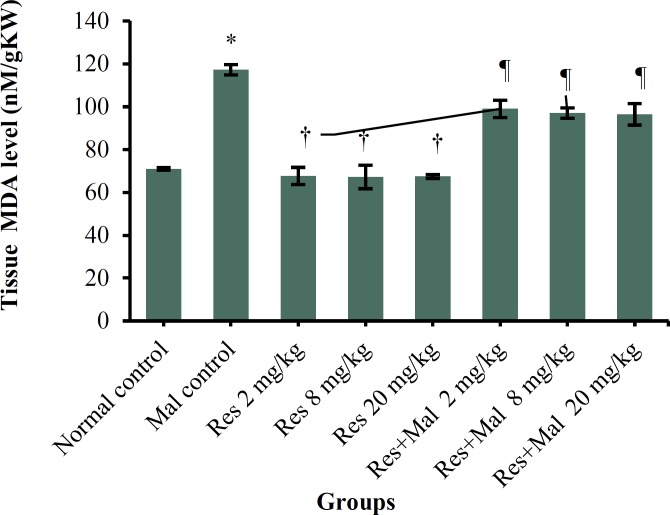
Comparison of testis MDA level between groups.

***MDA levels: ***Serum levels of MDA showed a significant increase in the Malathion control group compared to the normal control group (p < 0.001). Also, a significant decrease in MDA levels was showed in in all Resveratrol and Resveratrol + Malathion groups compared to the Malathion control group (p < 0.001) while had no significant effect on the levels of MDA in all Resveratrol groups compared to the normal control group (p > 0.05) (Figure 5).

*Significant decrease compared to the normal control group (p < 0.001). † Significant increase compared to the Malathion control group (p < 0.001). ‡Significant increase compared to the Malathion control group (p < 0.001). Rse, Resveratrol; Mal, Malathion.

***TAC levels: ***The results of measured TAC levels in the study groups showed a significant decrease in the Malathion control group compared to the normal control group (p < 0.001). 

* P < 0.001 compared to the normal control group. † P <0.001 compared to the Malathion control group. ¶p < 0.001 compared to the Malathion control group. MDA, malondialdehyde; Rse, Resveratrol; Mal, Malathion.

**Figure 6:**
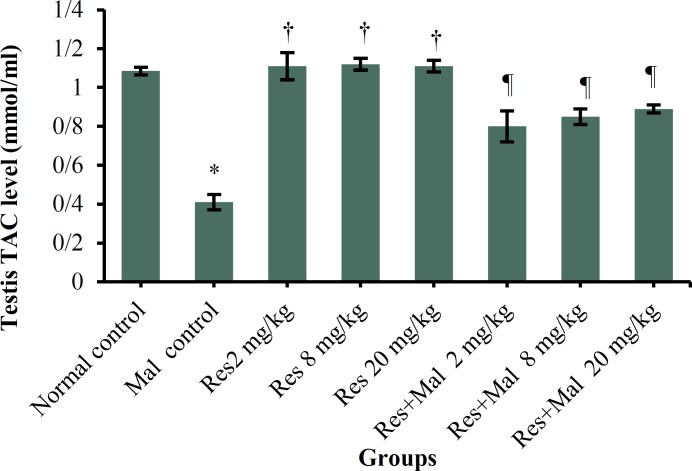
TAC level change in the male rats.

Also, a significant increase in TAC levels was showed in in all Resveratrol and Resveratrol + Malathion groups compared to the Malathion control group (p < 0.001) while had no significant effect on the levels of TAC in all Resveratrol groups compared to the normal control group (p > 0.05) (Figure 6).

* P < 0.001 compared to the normal control group. † p < 0.05 compared to Malathion control group. ¶ p < 0.05 compared to the Malathion control group. TAC, Total Antioxidant Capacity; Rse, Resveratrol; Mal, Malathion.

## Discussion

The World Health Organization (WHO) has reported 20,000 people dies annually due to poisoning with pesticides and 3,000,000 people suffer from nonlethal poisoning with pesticides. This number is so increasing that annually more than 700,000 people experience the chronic effects of contact with pesticides (22).Organophosphates have the highest effects on the immune system and the reproductive system. They can disturb male fertility through various ways such as direct impairment of cell structure and interference with biological processes (23). The findings of the current research suggested that Malathion administration had adverse and destructive effects on testis histology and sperm parameters, oxidant-antioxidant imbalance as well, and increase in testosterone hormone level. On the other hand, Resveratrol as a natural flavonoid relief the diverse effects of Malathion administration, obviously in male reproductive parameter. It also, recovers the cell damage offering by MDA decreasing and histology evaluation and the rate of oxidation (by calculating the amount of TAC). The current study results also showed that Resveratrol is able to reduce lipid peroxidation (decreased MDA) and increase anti-oxidant capacity (increased TAC) of testis tissue, thus it is reducing oxidative stress. Consistent with these findings, a large body of studies has shown anti-oxidant properties of Resveratrol (13, 15). Resveratrol apparently prevents the formation of lipid peroxidation induced by tert-butyl-hydroperoxide in sperm. Resveratrol is also a lipophilic molecule that is able to inhibit the production of lipid peroxidation via Fenton reaction (24). Thus, it appears that Resveratrol with its anti-oxidant properties could reduce MDA and increase TAC in the treatment groups by inhibiting the production of reactive oxygen species. Present study also indicated the recovery effect of Resveratrol on some male reproductive parameter as well as decreasing the oxidative stress by showing declining of MDA. Since sperms lose a large amount of their cytoplasm during spermatogenesis (lack of antioxidant systems), they seem to have higher sensitivity to elevated ROS than somatic cells (13). The first outcome of ROS attack to membrane structures can be cellular peroxidation in the cell membrane and organelles (4). Use of antioxidants such as resveratrol to eliminate toxic materials and free radicals from the cell surroundings can inhibit lipid peroxidation, thereby maintaining the biochemical structure of cells (25). The findings of Nahid et al. were in line with the results of the present study in that administration of Malathion significantly reduced catalase and serum total antioxidant level, increased lipid peroxidation, malondialdehyde and spermatogenesis damage, decreased the height of germinal epithelium, and reduced the number of primary spermatocytes in male rats compared to control group (26). The results of the present study showed that all sperm parameter in the Malathion control group reduced significantly compared to the normal control group. In Resveratrol and Malathion + Resveratrol groups, a significant increase was observed in all sperm cells parameter compared to the Malathion control group. Spermatogenesis is a highly complex process that is influenced by numerous factors, leading to infertility and reduced infertility in people (20). One of these factors is oxidative stress, which is induced by accumulation of ROS due to an imbalance between oxidant and antioxidant systems (17). ROS can affect DNA and RNA synthesis in the sperm cell and inhibit their mitochondrial functioning (18). Malathion-induced oxidative stress seems to disrupt the cell division and differentiation of sperms so that a number of spermatogonia are impaired on the base membrane and the number of primary and secondary spermatocytes, spermatids, and mature sperms is reduced (5). The results of Aitken et al. confirmed the findings of the present study in that oxidative stress disrupted spermatogenesis and led to defective gametes with remodeled chromatin vulnerable to the attack of free radicals and caused a reduction in the number of spermatogonia, spermatocytes, spermatids, and spermatozoa (27). Reduced number of sperms in the Malathion group might be due to the direct increase of oxidative stress-induced lipid peroxidation, which might have altered the natural properties of the membrane and consequently resulted in the loss of sperms transmitted to the epididymis and present in the epididymis (5). On the other hand, high levels of ROS causes mitochondrial impairment and consequently release of proapoptotic proteins in the intermembrane space, activation of caspases, reduction of ATP synthesis, elevated release of ROS, increased concentration of intracellular calcium, and release of calcium from mitochondria into cytosol, which in turn may lead to activation of apoptosis process (28). The findings of Selmi et al. were in agreement with the results of the current study, indicating that oral administration of Malathion significantly decreased the testis and body weight, sperm count, motility, and viability, and normal sperm morphology and increased sperm DNA damage in comparison with control group (5). Elevated free radicals can lead to impairment of Sertoli cells and destruction of cytoplasmic bridges via loss of epithelial cells, thereby decreasing the sperm count and sperm cell deformity (29). Resveratrol seems to have inhibitory effects on free radicals, to possess antioxidant properties, and to increase the number of anti-oxidative enzymes. The antioxidant ability of this polyphenol depends on the properties of its polyphenolic hydroxyl groups.(13) Resveratrol can exert its effects via a mechanism involved in the expression of oxidative phosphorylation genes and mitochondrial biogenesis (30). Resveratrol is also able to stabilize the blood-testis barrier and protect sperm DNA against the oxidative stress induced by free radicals (13). Reval et al. reported resveratrol could inhibit apoptotic induction and DNA damage against benzo[a]pyrene-induced oxidative stress in sperm, confirming the results of the present study (31). The present research showed a significant decrease in testosterone level in serum blood and diameter of seminiferous tubules in the Malathion group compared to the normal control group. Moreover, resveratrol significantly elevated testosterone level and height of the germinal layer of seminiferous tubules in all groups receiving Malathion plus resveratrol in comparison with the Malathion control group. Organophosphates can disrupt the expression of steroidogenic acute regulatory protein (StAR). This protein is a determinant of biosynthesis of steroids such as testosterone, and organophosphates directly disturb steroidogenesis in leydig cells by disrupting their expression (32). The results of Maliji et al. confirmed the findings of the current research in that administration of diazinon five days per week for a period of one month significantly elevated interleukin-1 and reduced testosterone in rats (33). In addition, it seems that organophosphates increase ACTH and cortisol. Increased ACTH and cortisol can inhibit the activity of hypothalamic-pituitary-gonadal axis, thereby disrupting the spermatogenesis process (34). Considering its potent antioxidant properties, resveratrol has positive effects on hypothalamic-pituitary-gonadal axis, testosterone level, and sperm production and motility. Furthermore, resveratrol is able to reduce apoptosis in germinal cell (35). Apparently, elevated ROS due to administration of Malathion increases lipid peroxidation, which in turn induces atrophy in the germinal layer thickness of seminiferous tubules (36). Salahshoor et al. showed a reduction in the epithelial volume of seminiferous tubules due to oxidative stress, which was in line with the findings of the present study (20). Resveratrol seems to protect lipids against peroxidation, prevent testicular oxidative stress, and play a role in the production of testicular steroids (13). The findings of Bitgul et al. were also in agreement with the results of the current study, indicating that oxidative stress impaired the germinal layer of seminiferous tubules compared to control group and resveratrol improved the germinal layer height of seminiferous tubules, reduced GSH and MDA, and elevated testosterone in groups exposed to oxidative stress (37). The present study showed that Malathion-induced male reproductive damage in rats could be reduced by plant antioxidants such as Resveratrol. Therefore, according to the foregoing, Resveratrol can improve the some male reproductive dysfunction, which has been caused by Malathion-induced toxicity considering its antioxidant properties.

## Conclusion

The outcomes of this study demonstration that Malathion can produce defects in some of male reproductive parameters and that a Resveratrol has an antioxidant and defending effect. It was revealed to growth the quality some spermatozoa and improved the normal morphology, sperm cell viability, germinal layer seminiferous tubules height, TAC, motility and count and reduce testis MDA level. Resveratrol could be valuable for the treatment of infertile men to enhancement male fertility. The antioxidant properties of Resveratrol could be a main reason for its optimistic outcome on reproductive parameters. Supplementary studies are essential to explain its careful mechanism of action.
